# Retraction: Naringin Alleviates Diabetic Kidney Disease through Inhibiting Oxidative Stress and Inflammatory Reaction

**DOI:** 10.1371/journal.pone.0192465

**Published:** 2018-02-01

**Authors:** The PLOS ONE Editors

The *PLOS ONE* Editors retract this publication [1] due to ethical concerns raised post-publication.

After publication of this article, the corresponding author notified *PLOS ONE* that this work had been submitted and published without his knowledge or consent, and that the email address listed on the article was not his own. During subsequent discussions with the authors and the institution, it was confirmed that the manuscript had been submitted without the corresponding author’s knowledge by one of the authors using a third-party that provided editing services.

In addition, the ethics statement included in the article is false. The ethics statement in the Methods section states, “The experiments were approved by the Institutional Animal Care and Use Committee of China Medical University.” However, the authors did not obtain ethical approval for the reported animal experiments. Hence, this work does not comply with *PLOS ONE*’s policy on animal research.

These issues were investigated by the First Hospital of China Medical University Professor Committee, who confirmed the above concerns.

In light of the ethical concerns raised, and in line with the institution’s recommendation, the *PLOS ONE* Editors retract this article.

The journal editors contacted all co-authors on the manuscript by email. QW made the initial request for retraction; all of the authors agreed with the retraction.
